# Long-Term Effects of the COVID-19 Pandemic: Emotional Regulation, Psychological Symptoms, and College Adjustment

**DOI:** 10.3390/ijerph22111731

**Published:** 2025-11-15

**Authors:** Barbara M. Gfellner, Ana I. Cordoba

**Affiliations:** 1 Department of Psychology, Brandon University, Brandon, MB R7A 6A9, Canada; 2 Faculty of Psychology, University of Valencia, 46010 Valencia, Spain; ana.cordoba@uv.es

**Keywords:** COVID-19 pandemic, emotional regulation, psychological symptoms, sex differences, college adjustment, culture, emerging adults

## Abstract

The COVID-19 pandemic was responsible for an unprecedented increase in psychological problems among post-secondary students worldwide. Drawing on data from a repeated cross-sectional (RCS) project, this study investigated changes in psychological symptoms, emotional regulation (cognitive reappraisal and emotional suppression), and academic, social, and personal–emotional college adjustment, and associations between these variables among students in two countries during the phases of lockdown (2021), lifting of restrictions (2022), and the endemic phase (2023). University students in Canada (n = 1014) and Spain (n = 447) completed online surveys during these periods. Students in both countries reported significant declines in perceived COVID-19 stress across the pandemic phases. In comparison with pre-pandemic rates, elevated psychological symptoms remained constant. There were some country differences, but sex differences were consistent. Psychological symptoms mediated the association between cognitive reappraisal and the adjustment measures among Canadian students during each pandemic period. Alternatively, they mediated the linkages of maladaptive emotional suppression with academic, social, and personal–emotional functioning of Spanish students at every phase, but only during the lifting of restrictions and the endemic phase for Canadian students. The results indicate the complexity of country and context in the role of emotional regulation during uncontrollable conditions and provide directions for intervention in stressful situations, including adjustment to university and future disastrous environmental events.

## 1. Introduction

The SARS-coronavirus (COVID-19) was declared a global pandemic by the WHO on 11 March 2020 [1]. It was responsible for unprecedented deaths, widespread personal, social, and economic disruption, and elevated mental health problems [2,3,4]. Extreme societal restrictions came into effect worldwide as a public health measure. Lockdowns that included school closures, working from home, and confined social interaction continued through the second year of the pandemic until vaccines became available. However, countries were not consistent in the extent of implementing restrictions [5]. The lifting of restrictions occurred during the third year, with personal safety controls maintained. On 5 May 2023, the WHO reclassified the COVID-19 pandemic as endemic [6], and what became known as “the new normal” ensued [7].

The onset of the COVID-19 pandemic raised awareness of mental health issues throughout the world [8,9,10], with a threat of undetermined long-term repercussions, especially among those most vulnerable [11,12,13]. However, the greatest incidence of psychological distress was among emerging adults (EAs), defined as young people between 18 and 29 years of age [14] with heightened rates for university students [15,16,17,18,19,20,21,22]. This was due to the disruption of developmental tasks that involve transitions in personal growth, including emotional and financial independence from family, educational goals, future planning, and forming romantic relationships. College students experienced abrupt university closures, the transition to distance online or hybrid education, and many moved back to living with parents, interrupting their emotional independence and social relationships fundamental to identity development [14]. The impact exacerbated psychological difficulties that typically appear or increase during this life period [21,23,24].

Numerous studies underscore the acceleration of mental health problems among university students during the first year of the pandemic relative to pre-COVID-19 rates [25,26,27,28,29]. Although most research was conducted during this time frame [15], elevated rates of psychological difficulties have been found to persist in the second wave [30,31,32], especially during lockdown [33,34].

Psychological distress, including stress, anxiety, depression, PTSD, sleep disturbance, and other diagnosable disorders, has been associated with individuals’ experience and perceptions of COVID-19 stress [17,35]. As with earlier research [36,37], women’s mental health problems have surpassed those of men during the pandemic [38,39]. However, more subtle differences may be associated with the role of culture and contextual factors, including how people process affect and react and cope with stressors during the pandemic [20,40,41,42,43].

Emotional regulation (ER) refers to the way individuals experience, express, and control their emotions in stressful and everyday situations [44,45]. Cognitive reappraisal (CR) and expressive (emotional) suppression (ES) are two well-defined and most frequently studied ER strategies with adaptive and maladaptive outcomes for mental health and well-being [44,46]. CR involves changing the way one interprets a stressful situation, which alters its meaning to alleviate negative impact. ES entails inhibiting ongoing emotion-expressive behavior with the potential of intensifying negative affect. Individuals who use CR tend to experience more positive and fewer negative emotions, while those who use mostly ES experience more negative emotions [47]. Considerable research shows that CR is associated inversely with psychological symptoms and positively with well-being, while ES is related to poor mental health [48,49,50,51,52,53]. Indeed, adaptive ER strategies function as protective factors in psychological adjustment and well-being [54].

General findings are consistent for ER in relation to psychological distress during the pandemic [55,56,57,58,59,60,61,62]. Most studies were conducted during lockdown when extreme confinement eliminated regular routines (face-to-face academic activities and social interactions with peers) relevant to an EA’s personal development [14,63]. A study of Italian university students found that adaptive ER and resilience were protective against distress from adverse lockdown conditions [64]. Similarly, CR buffered the impact of pandemic-related stress on internalizing symptoms of young adults in nine countries [65]. Another cross-cultural study of adults during lockdown reported higher levels of anxiety, increased use of maladaptive ER, and less resilience in the Polish sample [42]. Alternatively, despite elevated stress, Spanish respondents used more adaptive ER and were more resilient. These discrepant findings were attributed to the extent to which the countries differed in terms of individualism/collectivism. Although Spain is considered an individualistic country [66], its Mediterranean roots strongly value relatedness, family connection, group cohesiveness, and social responsibility. In comparison, Poland is individualistic in its emphasis on self-interest, self-determination, and a need to define oneself as different from others. In addition, women and younger people were at greater risk for psychological problems in Spain.

Although women typically engage in less ES than men, the relationship with psychopathology is the same [10,45,63,67]. According to Park et al. [63], the decreased use of ES among women is due to factors consistent with effects of the female gender (e.g., communalism, empathy, social support) promotive of more adaptive ER. These characteristics are also basic to collectivistic cultures [66,68].

Bronfenbrenner and Morris’s [69] ecological theory provided the framework to conceptualize potential differences in the role of context, that is, macroenvironmental changes, notably confinement restrictions and changes associated with the evolution of the pandemic. Drawing on data from a repeated cross-sectional (RCS) project, this study investigated students’ perceptions of pandemic stress, psychological symptoms, ER, and adjustment to university over the last three years of the pandemic (lockdown, LR, and endemic phase) with students in Spain and Canada.

Earlier pre-pandemic research [70,71] found greater resilience among Spanish than Canadian university students in terms of advanced psychosocial maturity despite elevated distress with personal identity issues. Further study reflected the resilience of Spanish students associated with the contextual disturbance of high youth unemployment in comparison with stable employment conditions among students in Canada [72]. In the current study, societal restrictions during the pandemic phases provide a series of unprecedented disruptive contexts expected to be reflected in students’ perceptions of COVID-19 stress, psychological distress, ER, and college adjustment. According to Hofstede’s [66] cultural dimensions theory, Canada is among the highest countries with an individualistic culture. As expected, an assessment of empathy and social perspective taking in 66 cultures found that Spain had elevated placement on these collectivistic characteristics in comparison with Canada [68]. Other related collectivistic qualities include conscientiousness and a strong sense of responsibility toward others and the community [73,74]. In the Palomera et al. [42] study, these cultural attributes were a protective factor that reflected Spanish adults’ resilience in the use of adaptive ER when dealing with pandemic stressors in comparison with the more individualistic Polish sample.

Collectivism has been positively associated with adherence to confinement restrictions and reduced psychological distress [62]. In other words, a strong sense of belonging, community, and interdependence with priority placed upon common needs, norms, and goals is reflected in compliance as a sense of security in national public health directives. This is seen in a sense of self-efficacy that the group will work together and act to protect themselves, and those protective processes are coordinated [75]. In a recent study, Xiao et al. [62] reported greater psychological symptoms and use of ES among Chinese students who attended university without lockdown as compared with similar students in the same community under lockdown conditions. In this situation, students without lockdown did not have the protection of institutional directives to support their collective concerns. Alternatively, the individualistic focus on personal autonomy, goals, preferences, and separation from others is associated with greater psychological distress [76].

Following earlier studies [70,71], greater use of adaptive ER would be expected among students in Spain than those in Canada. These pre-COVID-19 studies found differences between the countries in psychological distress. Nevertheless, the current study examined country and sex differences for consistency with prior research and to extend findings across the pandemic phases. In addition, psychological symptoms were investigated as a mediator in the association of ER with adjustment to university, an important indicator of functional well-being among university students. In the pre-COVID-19 cohort, social adjustment was greater among Spanish than Canadian students, and the groups did not differ on academic or personal–emotional functioning [70]. Consistent with Crede and Neihoester [77], women scored lower on personal–emotional adjustment than men.

In this study, we investigated the ER strategies of cognitive reappraisal (CR) and emotional suppression (ES) in relation to psychological problems and adjustment to university during the last three years of the pandemic. Our repeated cross-sectional (RCS) project on the transition to university with students in Canada and Spain provided the data.

During the second wave of the COVID-19 pandemic, Canada was in lockdown with severe restrictions. Spain utilized a hybrid educational condition, with half of the students alternating between weekly classes in-person and by distance, with social activities confined. The next academic year involved the lifting of public health restrictions with the availability of vaccines. This was followed by the WHO reclassification of the pandemic to endemic status in the next year. Our objective was to investigate how contextual changes over the course of the pandemic were associated with students’ perceived pandemic stress, ER (adaptive and maladaptive coping), psychological symptoms, and functioning at college. The predictions were as follows.

1. As indicated in the lessening of confinement restrictions over the course of the pandemic, declines were expected in perceived COVID-19 stress and psychological symptoms across the last two phases of the pandemic for students in each country.
2. As indicated in pre-COVID-19 studies with earlier cohorts in the RCS project [70,71], students in Canada were expected to score higher for perceived pandemic stress, psychological symptoms, and emotional suppression (ES), and lower on social adjustment in comparison with students in Spain across the pandemic phases.
3. Based on research [38,39,45,63,77], women were expected to score higher than men in their perceptions of COVID-19 stress, psychological problems, and lower in maladaptive RE (ES) and personal–emotional adjustment.
4. Psychological symptoms were predicted to mediate the linkage between ER and the college adjustment variables. Based on associations between ER and mental health [44,46], the indirect effect of psychological symptoms was expected to enhance adjustment in relation to cognitive reappraisal (CR) and to subvert adjustment in relation to emotional suppression (ES). It is unclear how this would be affected by contextual conditions of the COVID-19 phases and by country.

## 2. Materials and Methods

### 2.1. Procedure

Data were collected in a repeated cross-sectional (RCS) project among university students in Canada and Spain in 2021, 2022, and 2023. These periods coincided with pandemic phases of lockdown, lifting restrictions (LR), and the endemic phase. On each occasion, students completed an online survey in their respective languages. In each country, students were invited by instructors in their classes to complete a survey available on their class website. Students were awarded a bonus point toward their final grade in the course as a gratuity for participation. The standardized measures have been used consistently in the RCS project. Initially, items were translated from English to Spanish, tested for understandability, and subsequently back-translated for consistency. The study received ethical approval from the Brandon University Research Ethics Committee Certificate #20032-2.

### 2.2. Participants

Overall, 1481 students, 17 to 29 years of age, completed an online survey, including 1014 in Canada and 437 in Spain. Table 1 provides a complete description of the sample by country and pandemic phase.

### 2.3. Measures

**Perceived COVID-19 stress** was measured by a single item, “On a scale ranging from 1 to 10 where 10 represents “Extremely Stressful” and 1 represents “Not at All Stressful”, what was the highest level of stress you experienced during the COVID-19?”.

**Emotional Regulation** was measured with the cognitive reappraisal (CR) and emotional suppression (ES) scales of the Emotion Regulation Questionnaire (ERQ) [44]. The ERQ consists of 10 items: 4 for CR (e.g., “I control my emotions by changing the way I think about the situation I am in.”) and 6 for ES (e.g., “I control my emotions by not expressing them.”). Items are rated on a 7-point scale (“strongly disagree” to “strongly agree”) to indicate the extent to which they apply to the respondent. Psychometrics are seen in Peerce et al. [78] and Cabello et al. [79] for Spain.

**Psychological symptoms** were indexed by the Counseling Center Assessment of Psychological Symptoms (CCAPS) [80,81], a screening instrument to address the mental health needs of university students. It has widespread use for clinical screening, assessment, treatment planning, outcome monitoring, and evaluation in numerous universities in North America and other countries. The CCAPS-34 short form consists of 34 items rated on a 5-point scale from 0 (“not at all like me”) to 4 (“extremely like me”) to indicate the extent to which the symptom has been experienced in the past two weeks. The CCAPS-34 scales include depression, generalized anxiety, social anxiety, academic distress, eating concerns, hostility, and substance/alcohol abuse. This study used the Distress Index (CCAPS-DI), which provides a composite score of 20 psychological symptoms [82]. Psychometric properties are available in the CCAPS user Manual [81,83,84] and a recent psychometric analysis [85].

**The Student Adjustment to College Questionnaire (SACQ**) [86] is used extensively with university students as a screening tool by counseling services and in research. It assesses students’ academic, social, and personal–emotional functioning at university. The short form [87] consists of 28 items that are rated on a 9-point scale from “does not apply to me at all” to “applies very closely to me”, with a high score indicating adjustment. Crede and Neilhorster [77] provided a comprehensive review; psychometrics are available in the manual [86] and for Spanish students [88].

### 2.4. Data Analysis

**Figure 1:**
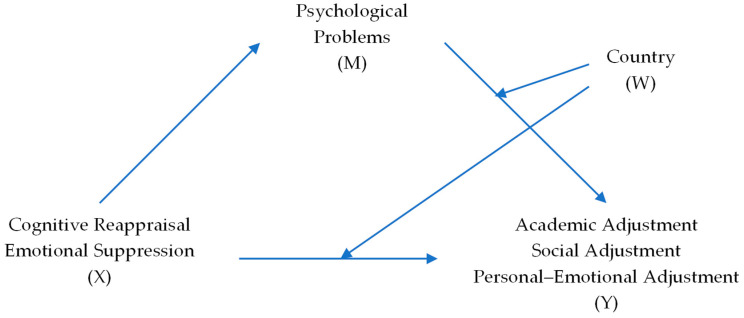
Conceptual model of second stage moderated mediation with emotional regulation (X) variables as the antecedents of adjustment (Y), with psychological symptoms (M) as the mediator, country (W) as the moderator, and sex as the covariate.

The sample characteristics by pandemic phase and country are given in Supplementary Table S1. Descriptive statistics were conducted for each phase of the pandemic, and ANOVAs were run for country x phase and country x sex by phase. Correlations were computed between the study variables by country for each phase, respectively. Hayes [89] PROCESS macro version 3 for SAS was used for the moderated mediation analyses. This macro provides analyses of direct and indirect effects in mediated, moderated, and moderated mediation models. Decisions about significant effects are made with bootstrap confidence intervals [89]. Figure 1 illustrates the conceptual model for the second stage of moderated mediation of the effects of the predictor ER (X) on the SACQ outcome variables (Y) with psychological symptoms/CCAPS (M) as the mediator, country (W) as the moderator, and sex as the covariate. This model was generated for the ER predictors and the college adjustment outcome variables for each pandemic phase, respectively.

## 3. Results

The first set of analyses examined country differences in the study variables over the three pandemic periods. The results for time by country are shown in Table 2. As predicted (H1), students’ ratings of perceived COVID-19 stress experienced across the pandemic periods decreased significantly from lockdown to LR to the endemic phase for those in Canada (F 2, 1019) = 20.91, *p* < 0.0001, eta = 0.04) and did not achieve significance among Spanish students (F 2, 433) = 2.51, *p* = 0.08, eta = 0.01). There were no significant differences in psychological symptoms over the pandemic phases among students in Canada (F 2, 952) = 1.58, *p* = 0.207, eta = 0.002), or those in Spain (F 2, 418) = 0.04, *p* = 0.964, eta = 1.7650 × 10^−4^). An increase in social adjustment scores (F 2, 1048) = 14.05, *p* < 0.0001, eta = 0.03) was seen from lockdown to LR for Canadian students. Hypothesis 1 was partially supported for Canadian students with a lessening of perceived COVID-19 stress over the course of the pandemic.

Country x sex ANOVAs were then run for each pandemic phase. As seen in Table 2 (left side) for the main effects of country by phase, Canadian and Spanish students’ perceived COVID-19 stress ratings did not differ significantly at each phase of the pandemic. However, during lockdown, Canadian students reported greater psychological symptoms and ES scores, while Spanish students scored higher on academic and social adjustment. Alternatively, during LR and the endemic phase, students in Canada continued to indicate greater ES and lower social adjustment scores than their Spanish cohorts. H2 was partially supported in terms of (a) higher psychological symptoms for students in Canada and higher academic adjustment for those in Spain during lockdown, and (b) higher ES for Canadian and social adjustment for Spanish students at each phase.

As there were no significant country x sex interactions, the main effects of sex by COVID-19 phase are reported. Taken together, overall sex differences for each of the three phases are shown on the right side of Table 2. As expected, during lockdown, women indicated elevated perceived COVID-19 stress, psychological symptoms, and lower ES and personal–emotional adjustment scores than men. During LR, these differences remained significant with women scoring higher on the psychological distress variables (perceived COVID-19 stress, psychological symptoms, and lower personal–emotional adjustment). Subsequently, during the endemic phase, elevated psychological distress persisted for these measures and for lower ES and greater academic adjustment. Hypothesis 3 was supported in terms of greater elevated psychological distress for women at each phase. However, women scored lower than men on ES during LR and the endemic phase, and they scored higher on academic adjustment during the endemic phase.

Table 3 shows the correlations between the study variables by country and pandemic phase. As expected, perceived COVID-19 stress related to psychological symptoms and PE adjustment on each occasion for students in both countries. Similarly, correlations were consistent between CCAPS and the SACQ variables. However, in some countries, by the pandemic phase, differences were seen in associations with the ER measures. During lockdown, CR correlated with CCAPS for students in Canada, and ES correlated with CCAPS for those in Spain. The associations were in the opposite direction, but as expected for the respective variables among students in both countries at LR. The pattern of correlations was similar to the lockdown during the endemic phase. Alternatively, on every occasion, CR correlated with the SACQ variables for Canadian students, and for those in Spain, only with academic adjustment at each phase and personal–emotional adjustment at the endemic phase (consistent with ES and CCAPS at LR). ES correlated significantly with social adjustment for Canadian students during lockdown and the endemic phase, and all SACQ variables at LR. In contrast, for Spanish students, associations were as expected for all SACQ variables at lockdown and for academic and PE adjustment during the LR and endemic phase.

The next set of analyses used the Hayes [89] process app to examine the second-stage moderated mediation as shown in Figure 1 for the conceptual model. Separate models were generated for the two ER predictors (X) and the three SACQ outcome variables (Y) with psychological symptoms, the mediator (M), country, the moderator (Y), and sex the covariate for each pandemic phase.

The conditional direct effect was significant for ES in relation to social adjustment of Canadian students during lockdown (B = −0.1705, SE = 0.0555, t = −3.07, *p* < 0.002, 95% CI = −0.2796 to −0.0614), through LR (B = −0.1106, SE = 0.0401, t = −2.756, *p* < 0.006), and the endemic phase, (B = −0.1328, SE = 0.0533, t = −2.493, *p* < 0.01, 95% CI = −0.2374 to −0.0281). The use of ES among Canadian students was associated with poor social adjustment at university during each of these pandemic periods. In addition, the conditional direct effect of CR predicted academic adjustment for students in Canada (B = 0.0961, SE = 0.0430, t = 2.24, *p* < 0.02, CI: 0.0117 to 0.1805), at the endemic phase. These were the only conditional direct effects that attained significance. Supplementary Table S2 provides a complete description of the coefficients for predictors in the models.

Table 4 shows the conditional indirect effects of psychological symptoms in the linkages of ER with the SACQ variables by pandemic phase and country. During lockdown, LR, and the endemic phases psychological symptoms mediated the positive effect of CR for academic, social, and personal–emotional functioning for university students in Canada. Alternatively, psychological symptoms mediated the negative effect of ES on these adjustment variables for Spanish students over all pandemic phases and among students in Canada only during LR and the endemic phase. H4 was supported in the predicted associations of ER and behavioral outcomes as mediated by psychological symptoms. However, the countries differed in the role of ER during the pandemic phases.

## 4. Discussion

This study examined psychological distress (including perceived COVID-19 stress and psychological symptoms), emotional regulation, and college adjustment in relation to contextual changes during the last three COVID-19 pandemic epochs (lockdown, LR, and endemic phase) using RCS data among university students in Canada and Spain. These findings are among the first to report on students’ mental health and adaptive functioning over and beyond the course of the COVID-19 pandemic.

As expected, students perceived pandemic stress significantly decreased across these occasions as restrictions were reduced and eliminated. In an RCS study with Spanish students, Matud et al. [90] found no change in the number of pandemic stressors reported from lockdown in June 2020 and 3 months later, with lockdown still in effect. As with current findings, the number of stressors declined significantly two years after the first assessment when restrictions were removed.

In comparison with pandemic stress, both Canadian and Spanish students’ psychological symptom scores during lockdown were significantly elevated in comparison with pre-COVID-19 cohorts in the same RCS project [32]. Furthermore, these scores remained stable during LR and the endemic phase. In comparison, Matud et al. [90] reported declines in anxiety and depression over 2 years, equal to the time between lockdown and LR, in their Spanish sample. It is unclear how differences between the samples and contexts may account for the discrepancy with current results. Indeed, our findings resonate with earlier concerns echoed about the potential long-term effects of the pandemic on the mental health of young people [91,92]. Considering the progressive increase in psychological symptoms among university students in pre-COVID-19 cohorts [37,93], the pandemic-accelerated increase may reflect a “new norm” in mental health.

The increase in social adjustment among Canadian students at LR reflects the opening of universities and return to in-class attendance with preventive guidelines in place. It enabled young people to resume face-to-face interaction and regular activities at university in a controlled social setting. In comparison, Spanish students’ higher social functioning scores remained consistent over the phases. These students engaged in hybrid education that consisted of alternating class participation weekly, with half the students attending in-person classes and the other half remotely from home. Distancing and other safety regulations were in place at the university. This context enabled in-person interaction with teachers and classmates, which may be reflected in greater social adjustment in comparison with the complete university shutdown of students in Canada. In a review of twelve longitudinal studies from before to the first 6 months of the pandemic with youth in the US, the Netherlands, and Peru, Barendse et al. [30] found that affective disorder rates were associated with the severity of lockdown. Similarly, Henseke et al. [94] reported positive effects of less stringent restrictions, including increased social contact as well as fewer concerns about learning and the future, in a longitudinal study of British adolescents in the second wave of the pandemic. The opportunity for social engagement was a major resource for EAs during the pandemic [95]. However, in the current study, Canadian students’ social adjustment scores plateaued at LD and remained significantly below those of their Spanish contemporaries. Spanish students’ stable social adjustment may be reflected in their lower psychological distress relative to Canadian students. In addition, the collectivistic culture reflects characteristics, including social embeddedness and interdependence with others and groups, that underscore social engagement as a resource [95].

Similarly, Spanish students’ lower ES scores reflect fewer psychological difficulties and more adaptive functioning [46]. In comparison, Canadian students’ elevated ES scores are indicative of their greater psychological distress [46]. This would explain the differences in psychological symptoms between Canadian and Spanish students at each of the pandemic phases. The collectivistic aspects of Spanish culture that involve empathy, interdependence, common goals, and cohesion are a supportive resource and protective factor associated with positive well-being and adjustment [48,52,55].

Consistent with earlier research [70,71], the current results underscore the resilience of Spanish students in comparison with Canadian cohorts in terms of reduced psychological symptoms, less use of ES, and greater social adjustment at each of the pandemic phases (H2). Nevertheless, the overall residual elevated psychological distress during the endemic phase is substantial for students in both countries. According to Youn et al. [82], a score of 1.0 is the lower cut-off point for diagnostic severity on the CCAPS-DI. The CCAPS-DI scores for students in Spain (i.e., 1.46, 1.47, and 1.45) and for those in Canada (i.e., 1.73, 1.65, and 1.60) for the respective pandemic phases indicate moderate and elevated severity that would benefit from clinical intervention. In comparison, the pre-COVID-19 scores of an earlier cohort of students in Canada (M = 1.3, sd = 0.77) and Spain (M = 1.2, sd = 0.65) were significantly lower and different [32]. The current findings underscore an urgent need for greater attention to effective preventive and intervention strategies to circumvent the elevated psychological difficulties of EAs [15].

As predicted, (H3) women evidenced greater psychological distress than men at each phase of the pandemic. Consistent with pre-COVID-19 global trends [37], this was seen with COVID-19 stress [38,55,90,96], psychological symptoms [39,97], and low personal–emotional adjustment [77,79]. According to Auerbach et al. [37], sex differences are more prominent than country differences in the incidence of psychological symptoms. The impact across these pandemic periods reflects a significant disadvantage for women. Nevertheless, as predicted [45,46], there was less use of ES (significant during lockdown and the endemic phase) among women than men. Taken together, these results warrant greater attention of university counseling and supportive mental health services to the unique needs of women and men. Matta and colleagues [98] emphasized the need to address biopsychosocial factors, integrating gender into mental health care, to understand the risk of post-COVID persistent symptoms and inform preventive and therapeutic strategies.

The correlations between perceived COVID-19 stress with psychological symptoms, psychological problems, and personal–emotional adjustment underscore the continuing effect of the pandemic on Canadian and Spanish students’ mental health. Similarly predicted associations were consistent between psychological symptoms with the college adjustment measures at each phase for students in both countries.

The differential alignment of ER with psychological symptoms and the SACQ variables during lockdown for CR among students in Canada and ES for those in Spain may be explained in terms of cultural differences in collectivism–individualism [66]. According to ER researchers [99,100], individualistic cultures discourage ES as it counteracts self-focus and control that may lead to feelings of inauthenticity. Alternatively, collectivistic cultures encourage a certain amount of ES to maintain harmony, given the increased awareness and emphasis on congenial interpersonal interactions. Overall, ES is associated with maladjustment across cultures, but it is attenuated in collective cultures [101]. Cebello et al. [79] reported a lack of correlation between ES and negative affect in the Mediterranean countries (Spain and Italy). Similarly, in a recent study of 19 countries during COVID-19, Tamir et al. [40] found that associations between ES and psychological health tended to be more negative in individualistic countries and low or unrelated in collectivistic cultures.

Indeed, in the current study for Spanish students during each pandemic phase, ES correlated significantly with CCAPS and all SACQ measures at lockdown, but not with social adjustment at LR and the endemic phase. Alternatively, for Canadian students, the only significant correlation for ES was with social adjustment at lockdown, and correlations were as expected at LR and the endemic phase. Consistent positive associations were seen between CR with CCAPS and the SACQ measures during each phase for Canadian students. In contrast, for Spanish students, there was some consistency at LR, but the only significant CR correlation was with academic adjustment during lockdown and the endemic phase.

Taken together, correlations for the countries across the phases indicate some differences in the use of ES and CS among Spanish and Canadian students, respectively. The differential alignment of these strategies at lockdown appears to reflect cultural differences in response to the transition. Xiao et al. [62] found greater use of ES and psychological distress among Chinese students attending university in person as opposed to those in confined lockdown conditions. As noted previously, the protection of the university closure was explained as a sense of confidence and security attributed to societal regulations in the collective culture. In the current study, the countries reflect differences in the extent of confinement, that is, a total shutdown for Canadian students and a hybrid condition for those in Spain. ES related to psychological symptoms for Spanish students at lockdown, but not for those in Canada. Other studies indicated that differences in confinement conditions implemented by countries were variably associated with psychological distress [30,94].

Psychological symptoms significantly mediated linkages between ER and the college adjustment measures over the pandemic phases, as indicated in the correlations. There were distinct differences between students in the two countries during lockdown. Specifically, psychological symptoms positively mediated CR on each of the college adjustment measures of Canadian students. Alternatively, for students in Spain, psychological symptoms augmented the linkage between ES and the functional domains at university. These mediational effects persisted over the subsequent LR and endemic phases. In addition, for students in Canada, psychological symptoms amplified the association between ES and maladaptive college functioning, as with their Spanish cohorts. Although the results are consistent with ER (CR and ES) associations with adjustment [46,48,49,53], the differential alignment of effects reflects cultural differences [40].

The individualistic orientation is characterized by self-focus, autonomy, and personal control, so that when threatened by the uncertainty of the pandemic, these students may be motivated to achieve a way of understanding the situation. For Canadian students, the positive benefits of CR were mediated through lessened psychological symptoms. This coping strategy was maintained over subsequent phases of the pandemic as confinements were removed, thereby increasing individuals’ sense of control. During the latter two phases, the indirect role of psychological symptoms in the ES linkage with these outcomes is consistent with their individualistic culture [40,102].

An absence of CR among Spanish students may be explained by cultural factors. The collectivistic adherence to societal restrictions during the pandemic [76] reflects the importance of connection with others, especially family, the community, and conscientiousness [74,103]. In other words, the sense of interdependence, shared purpose, and responsibility during the unprecedented situation and confidence in national restrictions served as a protective factor against psychological distress [55]. This is consistent with the lower psychological distress of Spanish students in comparison with those in Canada. Alternatively, CR functions as a coping resource among Canadian students with an individualistic orientation during these changing pandemic contexts as opportunities for personal control increase [104].

There are several limitations of this research. First, the study is part of an RCS project that includes different cohorts of EA in the same context (student body) over time. It enabled data collection in a timely manner, given the immediacy of changes during the COVID-19 pandemic. Longitudinal follow-up of students would enable an evaluation of prospective changes over time. Nevertheless, Zetter et al. [105] compared RCS with panel studies during the early phase of COVID-19 and reported some subtle differences as well as advantages of cross-sectional research. According to Aknin et al. [15], the use of both methods allows researchers to draw more convincing conclusions. Second, the measure of perceived COVID-19 stress was indexed by a single item. The use of an abbreviated indicator is considered appropriate when dealing with time-constrained data collection [106]. Third, this study utilized the two most popular ER strategies [46]. The inclusion of more strategies would offer a broader perspective on the scope of students’ ER styles [40,48]. For example, social support is an ER style that is part of collectivism [66]. Fourth, the study relied on a quantitative self-report method that may be subject to social desirability. The use of data from other sources, such as parents and counselors, as well as qualitative reporting from students, would provide a broader view and more nuanced information. Finally, greater participation of women than men is a common problem and drawback in research with university students [107]. In this study, the high proportion of females may be exacerbated by the gender bias in the education career program of Spanish students and the sex distribution, in general, of introductory classes taken by students in the general arts and science program in Canada.

## 5. Conclusions

In conclusion, the results are among the first to highlight the enduring impact of the COVID-19 pandemic on psychological distress among university students in Canada and Spain. Consistent with pre-pandemic findings [70,71], the resilience of Spanish students was seen in significantly lower psychological symptom and ES scores compared to Canadian cohorts. Overall, females were disproportionately disadvantaged in comparison with males. Differences between the countries with psychological symptoms mediating in the ER linkages with adjustment at university warrant the inclusion of culturally sensitive ER intervention strategies. ER training is an expedient intervention in clinical, counseling, and medical health. Evidence-based approaches that focus on teaching ER skills are well-developed for clinical and non-clinical settings [46,78]. The findings have important implications in the understanding of uncontrollable events at the regional, national, and global levels (including future epidemics, wars, political dishevel, global recession, pervasive ramifications associated with climate change, and media coverage of disastrous events) that may vary in terms of duration and predictability. Ongoing research is required to monitor and address the implications for mental health of potential subsequent catastrophic events.

## Figures and Tables

**Table 1:** Demographics by COVID-19 pandemic phase and country.

	Lockdown		LR		Endemic Phase	
	Canada	Spain	Canada	Spain	Canada	Spain
N	288	149	357	155	428	143
**Age**	20.8 (2.8)	18.8 (1.6)	20.1 (2.6)	18.5 (1.0)	19.4 (2.4)	18.6 (1.7)
Female	75.4%	88.4%	79.8%	81.9%	73.4%	84.6%
**Marital Status**						
Single	93%	98%	93.8%	89%	95.3%	86.7%
Married/common law	7%	2%	5.9%	11%	4.7%	13.3%
Divorced/separated	-	-	0.3%	-	-	-
**Race/Background**						
White/Caucasian	71%	98%	57.6%	92.3%	58.9%	92.3%
First Nation, Metis, Indigenous	8.5%	-	12.1%	-	7.8%	-
Asian	11.9%	0.5%	10.4%	-	9.4%	-
Arab	-	1.5%	-	2.6%	-	2.1%
Black	8.6%	-	17.3%	-	3.1%	-
Mestiza	-	-	-	3.2%	0.5%	-
International	-	-	1.7%	-	-	-
Other	-	-	1.1%	1.9%	-	-
**Living Situation**						
Home with parents	59%	64.4%	34.3%	60.7%	43.9%	57.3%
Spouse/common-law partner	8%	2%	5.9%	-	5.2%	-
Roommates in house/apt	25.7%	28.9%	37.1%	-	32.6%	35%
Residence	2.4%	1.3%	8.2%	31.0%	9.6%	-
Alone	3.5%	2.0%	11.5%	8.4%	6.6%	6.3%
Grandparents, other family	1.9%	0.7%	3.1%	-	1.6%	1.4%

**Table 2:** Summary of the mean (SD) scores for country and sex by COVID-19 phase.

	Country				SEX			
	Canada	Spain			Female	Male		
	M (SD)	M (SD)	F-Value	eta	M (SD)	M (SD)	F-Value	eta
**LOCKDOWN**								
Perceived COVID-19 Stress	7.7 (2.5)	6.9 (2.4)	0.13		7.4 (2.2)	6.0 (3.0)	14.64 ****	0.03
Psychological Symptoms	1.73 (0.86)	1.46 (0.79)	4.99 *	0.01	1.71 (0.84)	1.31 (0.75)	13.27 ***	0.03
Cognitive Reappraisal	4.61 (1.2)	4.69 (1.0)	0.39		4.63 (1.1)	4.66 (1.2)	0.11	
Emotional Suppression	3.99 (1.4)	3.55 (1.3)	7.70 **	0.02	3.74 (1.4)	4.26 (1.4)	5.16 *	0.01
Academic Adjustment	6.09 (1.2)	6.56 (1.3)	4.62 *	0.01	6.26 (1.3)	6.17 (1.1)	0.63	
Social Adjustment	4.49 (1.3)	6.15 (1.4)	92.29 ****	0.07	5.03 (1.6)	5.08 (1.5)	1.52	
Personal Emotional Adjustment	4.41 (1.6)	4.60 (1.7)	3.29		4.27 (1.7)	5.24 (1.5)	24.72 ****	0.01
**LIFTING RESTRICTIONS**								
Perceived COVID-19 Stress	6.7 (2.5)	6.7 (2.3)	0.00		6.9 (2.4)	5.9 (2.6)	9.47 **	0.02
Psychological Symptoms	1.65 (0.86)	1.47 (0.63)	1.12		1.64 (0.82)	1.35 (0.70)	5.89 **	0.01
Cognitive Reappraisal	4.63 (1.2)	4.72 (1.0)	0.06		4.61 (1.1)	4.85 (1.8)	1.20	
Emotional Suppression	4.11 (1.3)	3.51 (1.4)	14.45 ***	0.02	3.85 (1.4)	4.17 (1.3)	1.96	
Academic Adjustment	6.27 (1.2)	6.68 (1.1)	3.45		6.42 (1.2)	6.29 (1.2)	2.29	
Social Adjustment	5.10 (1.5)	6.23 (1.2)	32.04 ****	0.06	5.41 (1.5)	5.58 (1.5)	0.29	
Personal Emotional Adjustment	4.52 (1.7)	4.64 (1.6)	0.04		4.45 (1.6)	5.01 (1.6)	6.42 **	0.01
**ENDEMIC**								
Perceived COVID-19 Stress	5.7 (2.8)	6.1 (2.5)	1.81		6.1 (2.6)	4.8 (2.8)	10.85 ***	0.02
Psychological Symptoms	1.60 (0.84)	1.45 (0.74)	1.63		1.63 (0.82)	1.31 (0.74)	6.99 **	0.01
Cognitive Reappraisal	4.61 (1.2)	4.70 (1.1)	0.90		4.61 (1.2)	4.70 (1.1)	0.51	
Emotional Suppression	4.22 (1.3)	3.33 (1.4)	22.21 **	0.04	3.85 (1.4)	4.44 (1.4)	9.56 **	0.02
Academic Adjustment	6.38 (1.1)	6.60 (1.2)	0.11		6.50 (1.1)	6.20 (1.2)	12.88 ****	0.02
Social Adjustment	5.05 (1.4)	6.10 (1.4)	33.12 ****	0.06	5.34 (1.5)	5.25 (1.4)	0.00	
Personal Emotional Adjustment	4.75 (1.7)	4.83 (1.7)	0.04		4.62 (1.7)	5.27 (1.5)	5.15 *	0.01

Notes. **** = *p* < 0.0001, *** = *p* < 0.001, ** = *p* < 0.01, * = *p* < 0.05.

**Table 3:** Correlations for the variables and alphas by pandemic phases and country ^1^.

**LOCKDOWN**	**1.**	**2.**	**3.**	**4.**	**6.**	**7.**	**8.**	**α**
1. Perceived COVID-19 Stress	-	0.29 ^a^	−0.08	−0.17 ^b^	−0.07	−0.15 ^b^	−0.30 ^a^	-
2. Psychological Symptoms	0.22 ^c^	-	−0.36 ^a^	0.06	−0.39 ^a^	−0.34 ^a^	−0.74 ^a^	0.92
3. Cognitive Reappraisal	0.18 ^c^	−0.09	-	0.10	0.19 ^b^	0.14 ^d^	0.24 ^a^	0.87
4. Emotional Suppression	0.10	0.39 ^a^	0.04	-	−0.06	−0.21 ^b^	0.02	0.81
6. Academic Adjustment	0.06	−0.47 ^a^	0.12	−0.18 ^d^	-	0.28 ^a^	0.34 ^a^	0.76
7. Social Adjustment	0.08	−0.51 ^a^	0.09	−0.29 ^b^	0.57 ^a^	-	0.26 ^a^	0.77
8. Personal–Emotional Adjustment	−0.16	−0.74 ^a^	0.07	−0.19 ^d^	0.44 ^a^	0.37 ^a^	-	0.75
α	-	0.91	0.82	0.76	0.84	0.87	0.83	
**LIFTING RESTRICTIONS**	**1.**	**2.**	**3.**	**4.**	**6.**	**7.**	**8.**	**α**
1. Perceived COVID-19 stress	-	0.26 ^a^	0.00	−0.04	−0.06	−0.00	−0.16 ^c^	-
2. CCAPS	0.20 ^b^	-	−0.18 ^b^	0.30 ^a^	−0.46 ^a^	−0.37 ^a^	−0.70 ^a^	0.93
3. Cognitive Reappraisal	−0.05	−0.17 ^d^	-	0.19 ^b^	0.17 ^c^	0.15 ^c^	0.16 ^c^	0.87
4. Emotional Suppression	−0.07	0.34 ^a^	0.11	-	−0.19 ^b^	−0.14 ^c^	−0.20 ^b^	0.77
6. Academic Adjustment	−0.05	−0.30 ^b^	0.21 ^c^	−0.12	-	0.35 ^a^	0.41 ^a^	0.77
7. Social Adjustment	−0.03	−0.17 ^d^	0.09	−0.09	0.52 ^a^	-	0.25 ^a^	0.83
8. Personal–Emotional Adjustment	−0.26 ^b^	−0.72 ^a^	0.19 ^c^	−0.22 ^b^	0.23 ^b^	0.37 ^a^	-	0.78
α	-	0.88	0.79	0.81	0.78	0.82	0.77	-
**ENDEMIC PHASE**	**1.**	**2.**	**3.**	**4.**	**6.**	**7.**	**8.**	**α**
1. Perceived COVID-19 Stress	-	0.17 ^b^	0.08	−0.01	−0.02	−0.03	−0.19 ^a^	-
2. CCAPS	0.17 ^d^	-	−0.23 ^a^	0.13 ^c^	−0.37 ^a^	−0.31 ^a^	−0.69 ^a^	0.93
3. Emotional Reappraisal	0.08	−0.05	-	0.20 ^a^	0.19 ^b^	0.11 ^d^	0.20 ^a^	0.86
4. Emotional Suppression	−0.08	0.24 ^b^	0.04	-	−0.08	−0.16 ^c^	−0.08	0.77
6. Academic Adjustment	−0.10	−0.34 ^a^	0.15	−0.15	-	0.27 ^a^	0.37 ^a^	0.73
7. Social Adjustment	0.03	−0.23 ^b^	0.01	−0.09	0.42 ^a^	-	0.31 ^a^	0.80
8. Personal–Emotional Adjustment	−0.23 ^b^	−0.74 ^a^	−0.01	−0.17 ^d^	0.29 ^b^	0.20 ^c^	-	0.78
α	-	0.92	0.77	0.80	0.79	0.85	0.80	-

Notes. ^1^ Correlations for the students in Canada are above the diagonals and below the diagonals for those in Spain; ^a^ = *p* < 0.0001, ^b^ = *p* < 0.001, ^c^ = *p* < 0.01, ^d^ = *p* < 0.05.

**Table 4:** Indirect effects of psychological symptoms in the ER linkage with college adjustment among Canadian and Spanish students during the pandemic conditions of lockdown, LR, and the endemic phase.

Predictor by Time and Country	Academic Adjustment		Social Adjustment		Personal–Emotional Adjustment	
	Effect (Boot SE)	95%CI	Effect (Boot SE)	95%CI	Effect (Boot SE)	95%CI
LOCKDOWN						
**Cognitive Reappraisal**						
Canada	0.1310 (0.0330)	0.0717 to 0.2011	0.1413 (0.0341)	0.0801 to 0.2132	0.3502 (0.0613)	0.2361 to 0.4746
Spain	0.0531 (0.0629)	−0.0611 to 0.1901	0.0606 (0.0721)	−0.0687 to 0.2316	0.1085 (0.1271)	−0.1318 to 0.8886
**Emotional Suppression**						
Canada	−0.0325 (0.0217)	−0.0785 to 0.0084	−0.0329 (0.0223)	−0.0807 to 0.0097	−0.0824 (0.0523)	−0.1885 to 0.0257
Spain	−0.2032 (0.0527)	−0.3028 to −0.1052	−0.2036 (0.0527)	−0.3142 to −0.0116	−0.4286 (0.0865)	−0.5862 to −0.2516
LIFTING RESTRICTIONS						
**Cognitive Reappraisal**						
Canada	0.0786 (0.0186)	0.0445 to 0.1181	0.0786 (0.0186)	0.0445 to 0.1181	0.1897 (0.0409)	0.1122 to 0.2755
Spain	0.0369 (0.0210)	−0.0014 to 0.0814	0.0369 (0.0212)	−0.0014 to 0.0814	0.1319 (0.0706)	−0.0051 to 0.2718
**Emotional Suppression**						
Canada	−0.0866 (0.0168)	−0.1227 to −0.0558	−0.0762 (0.0165)	−0.1125 to −0.0470	−0.2064 (0.0365)	−0.2828 to −0.0.1374
Spain	−0.0734 (0.0202)	−0.1191 to −0.0390	−0.0552 (0.0212)	−0.1009 to −0.0173	−0.2605 (0.0494)	−0.3631 to −0.1616
ENDEMIC PHASE						
**Cognitive Reappraisal**						
Canada	0.0718 (0.0207)	0.0339 to 0.1123	0.0740 (0.0216)	0.0349 to 0.1191	0.2155 (0.0517)	0.1175 to 0.3152
Spain	0.0221 (0.0317)	−0.0368 to 0.0906	0.0188 (0.0279)	−0.0327 to 0.0810	0.0739 (0.1007)	−0.1186 to 0.2748
**Emotional Suppression**						
Canada	−0.0502 (0.0182)	−0.0883 to −0.0148	−0.0472 (0.0183)	−0.0861 to −0.0143	−0.1436 (0.0510)	−0.2423 to −0.0432
Spain	−0.0690 (0.0301)	−0.1332 to −0.0187	−0.0578 (0.0310)	−0.1257 to −0.0062	−0.2366 (0.0753)	−0.3892 to −0.0848

## Data Availability

Data will be made available upon reasonable request to the first author.

## Supplementary Materials

The following supporting information can be downloaded at: https://www.mdpi.com/article/10.3390/ijerph22111731/s1, Table S1: Means (SD), range, skewness, kurtosis of the study variables by country and COVID-19 phase; Table S2: Summary of the Effects of Predictors by Pandemic Phase.
