# Supplementary material for: Long-Term Effects of the COVID-19 Pandemic: Emotional Regulation, Psychological Symptoms, and College Adjustment
Source: Int J Environ Res Public Health. 2025 Nov 15;22(11):1731. doi: 10.3390/ijerph22111731 (PMC12652499; doi:10.3390/ijerph22111731)
Supplement: Supplementary file 1 [file ijerph-22-01731-s001.zip › Supplementary Table S1 - Sample Mean SD Rande Skew Kurtosis.pdf]

Supplementary Table S1. Means (SD), range, skewness, kurtosis of the study variables by country and COVID-19 phase

Variables by Pandemic Phase	Canada				Spain			
	M (SD)	Range	Skewness	Kurtosis	M (SD)	Range	Skewness	Kurtosis
**Lockdown**								
COVID-19 Stress	7.25(2.5)	1-10	-0.9866	0.25197	6.90 (2.4)	1-10	-0.6747	-0.1200
Psychological Symptoms/CCAPS	1.73 (.86)	0-3.8	0.1428	-0.6223	1.47 (.79)	1-3.9	0.7845	0.3420
Cognitive Appraisal	4.61 (1.2)	1-7	-0.3461	0.2118	4.69 (.98)	2-7	-0.9871	0.0130
Emotional Suppression	3.99 (1.4)	1-7	-0.0123	-0.7409	3.55 (1.3)	1-6.5	0.0039	-0.6043
Academic Adjustment	6.10 (1.2)	2.5-9	-0.2488	-0.1648	6.56 (1.3)	2.4-9	-0.5163	0.3135
Social Adjustment	4.49 (1.3)	1.4-8	0.1649	-0.4225	6.16 (1.4)	1.7-9	-0.5811	0.5294
Personal-Emotional Adjustment	4.41 (1.6)	1-8.6	0.2371	-0.6027	4.60 (1.7)	1-8.5	0.1159	-0.7372
**Lifting Restrictions**								
COVID-19 Stress	6.67 (2.5)	1-10	-0.5685	-0.5217	6.69 (2.3)	1-10	-0.5825	-0.3800
Psychological Symptoms/CCAPS	1.65 (.86)	0-3.85	0.2950	-0.3875	1.47 (.65)	0.15-3.5	0.2439	-0.2629
Cognitive Appraisal	4.63 (1.2)	1-7	-0.1358	-0.0133	4.72 (1.0)	2-7	-0.2259	-0.2480
Emotional Suppression	4.11 (1.3)	1-7	-0.0504	-0.4706	3.51 (1.4)	1-6.8	-0.0159	-0.8022
Academic Adjustment	6.26 (1.2)	2.5-9	-0.2406	-0.3339	6.68 (1.1)	2.2-8.6	-0.9483	1.4774
Social Adjustment	5.10 (1.5)	1.2-8.6	-0.1588	-0.2385	6.23 (1.2)	1.8-8.4	-0.5491	0.2823
Personal Emotional Adjustment	4.52 (1.7)	1-9	0.1347	-0.2347	4.64 (1.6)	1.1-8.4	0.0930	-0.4507
**Endemic**								
COVID-19 Stress	5.70 (2.8)	1-10	-0.2148	-1.0305	6.14 (2.5)	1-10	-0.3092	-0.8459
Psychological Symptoms/CCAPS	1.60 (.84)	0-3.75	0.1718	-0.6086	1.45 (.74)	.10-3.2	0.1178	-0.7504
Cognitive Appraisal	4.61 (1.2)	1-7	-0.1588	0.1429	4.70 (1.1)	1.8-7.0	-0.1719	-0.2316
Emotional Suppression	4.22 (1.3)	1-7	-0.1604	-0.2922	3.33 (1.4)	1-6.8	-0.0832	-0.0765
Academic Adjustment	6.38 (1.1)	3.7-9	0.0113	-0.4539	6.60 (1.2)	2.2-9	-0.7652	1.2342
Social Adjustment	5.05 (1.4)	1.2-9	0.1253	-0.2217	6.10 (1.4)	1.4-9	-0.3962	0.1250
Personal Emotional Adjustment	4.75 (1.7)	1-9	0.0670	-0.4456	4.83 (1.7)	1.3-8.8	0.1889	-0.5010
