# Supplementary material for: Long-Term Effects of the COVID-19 Pandemic: Emotional Regulation, Psychological Symptoms, and College Adjustment
Source: Int J Environ Res Public Health. 2025 Nov 15;22(11):1731. doi: 10.3390/ijerph22111731 (PMC12652499; doi:10.3390/ijerph22111731)
Supplement: Supplementary file 1 [file ijerph-22-01731-s001.zip › Supplementary Table S2. Summary of effects of predictors by phase.pdf]

Table S2. Summary of the Effects of Predictors by Pandemic Phase

**Outcome**	Predictor	t-value	95%CI
**LOCKDOWN**			
	**Cognitive Reappraisal**		
CCAPS (M)	-0.1919 (0.037)	-5.26****	-0.26365 to -0.1202
Academic Adjustment	0.0748 (0.053)	ns	
Social Adjustment	0.0427 (0.060)	ns	
Personal-Emotional	-0.0115 (0.054)	ns	
	**Emotional Suppression**		
CCAPS (M)	0.1265 (0.030)	4.20****	0.0672 to 0.1857
Academic Adjustment	0.0002 (0.043)	ns	
Social Adjustment	-0.1571(0.048)	-3.27**	-0.2515 to -0.8626
PE Adjustment	0.0953 (0.435	2.19*	0.0097 to 0.1809
**LIFTING RESTRICTIONS**			
	**Cognitive Reappraisal**		
CCAPS (M)	-0.1225 (0.022)	-5.47****	-0.1665 to -0.0785
Academic Adjustment	0.0051 (0.038)	3.64***	0.0485 to 0.1617
Social Adjustment	0.0511 (0.037)	ns	
PE Adjustment	0.0363 (0.034)	ns	
	**Emotional Suppression**		
CCAPS (M)	0.1522 (0.019)	7.85****	0.1141 to 0.1902
Academic Adjustment	-0.0288 (0.026)	ns	
Social Adjustment	-0.0849 (0.033)	-2.55**	-0.1501 to -0.0196
PE Adjustment	0.0120 (0.030)	ns	
**ENDEMIC**			
	**Cognitive Reappraisal**		
CCAPS (M)	-0.1262 (0.030)	-4.19****	-0.1853 to -0.0670
Academic Adjustment	0.1020 (0.038)	2.68**	0.0272 to 0.1078
Social Adjustment	0.0307 (0.050)	ns	
PE Adjustment	0.0135 (0.045)	ns	
	**Emotional Suppression**		
CCAPS (M)	0.1127 (0.028)	4.09****	0.0585 to 0.1669
Academic Adjustment	-0.0255 (0.035)	ns	
Social Adjustment	-0.1060 (0.046)	-2.33*	-0.1953 to -0.0166
PE Adjustment	0.0003 (0.041)	ns	

Notes. CCAPS = psychological symptoms; **** = p < .0001, *** = p < .001, ** = p < .01, * = p < .05
